# Prognostic gene biomarkers for c-Src inhibitor Si162 sensitivity in melanoma cells

**DOI:** 10.55730/1300-0152.2678

**Published:** 2023-11-06

**Authors:** Seyhan TÜRK, Ayşegül YILMAZ, Ümit Yavuz MALKAN, Gülberk UÇAR, Can TÜRK

**Affiliations:** 1 Department of Biochemistry, Faculty of Pharmacy, Hacettepe University, Ankara, Turkiye; 2 Department of Medical Microbiology, Faculty of Medicine, Lokman Hekim University, Ankara, Turkiye; 3 Department of Hematology, Faculty of Medicine, Hacettepe University, Ankara, Turkiye

**Keywords:** Melanoma, c-Src inhibitor, si162, gene biomarker, chemotherapy

## Abstract

**Background/aim:**

Early detection and treatment are crucial in combating malignant melanoma. Src is an important therapeutic target in melanoma due to its association with cancer progression. However, developing effective Src-targeting drugs remains challenging and personalized medicine relies on biomarkers and targeted therapies for precise and effective treatment. This study focuses on Si162, a newly synthesized c-Src inhibitor, to identify reliable biomarkers for predicting Si162 sensitivity and explore associated biological characteristics and pathways in melanoma cells.

**Materials and methods:**

Primary melanoma cells (M1, M21, M24, M84, M133, M307, and M2025) were obtained from patients diagnosed with melanoma. Si162 cytotoxicity tests were performed using luminescent adenosine triphosphate detection and the half-maximal inhibitory concentration (IC_50_) values were calculated. Gene expression profiles were analyzed using microarray-based gene expression data. Differentially expressed genes between the resistant and sensitive groups were identified using Pearson correlation analysis. Gene coexpression, interactions, and pathways were investigated through clustering, network, and pathway analyses. Biological functions were examined using the Database for Annotation, Visualization, and Integrated Discovery. Molecular pathways associated with different responses to Si162 were identified using gene set enrichment analysis. The gene expressions were validated using reverse transcription-quantitative polymerase chain reaction.

**Results:**

The cells revealed significant differences in response to Si162 based on the IC_50_ values (p < 0.05). A total of 36 differentially expressed genes associated with Si162 susceptibility were identified. Distinct expression patterns between the sensitive and resistant groups were observed in 9 genes (LRBA, MGMT, CAND1, ADD1, SETD2, CNTN6, FGF18, C18orf25, and RPL13). Coexpression among the differentially expressed genes was highlighted, and 9 genes associated with molecular pathways, including EMT, transforming growth factor-beta (TGF-β) signaling, and ribosomal protein synthesis, between groups. Genes involved in dysregulated immune response were observed in the resistant group. The involvement of 5 genes (ADD1, CNTN6, FGF18, C18orf25, and RPL13) in Si162 resistance was confirmed through qRT-PCR validation.

**Conclusion:**

These findings contribute to our understanding of the underlying biological differences among melanoma cells and suggest potential biomarkers and pathways associated with Si162 response and resistance.

## 1. Introduction

Melanoma, a highly prevalent and deadly form of cancer, has shown a consistent increase in incidence over the past few decades. This trend is particularly notable in countries like Türkiye, where the rising number of skin cancer cases may be attributed to environmental factors, including the ongoing depletion of the ozone layer in the stratosphere (Daghan et al., 2014; Siegel et al., 2020). To effectively combat malignant melanoma, early detection and treatment are of paramount importance. While targeted therapies against components of the mitogen-activated protein kinase signaling cascade and immunotherapies targeting immunological checkpoints have improved patient outcomes, melanoma remains one of the most lethal cancers (Luke et al., 2017).

Among the potential therapeutic targets in melanoma, Src, a nonreceptor tyrosine kinase and the first proto-oncogene discovered in the human genome, has emerged as an important player. Overexpression of Src and its family kinases has been associated with accelerated progression of various cancer types, including melanoma, making the clinical development of Src-targeting drugs a critical challenge. Several studies have demonstrated the utility of Src inhibitors in the treatment of malignant melanoma, given the pivotal role of Src in the development of the disease and therapy (Cavenee et al., 1989; Paul and Mukhopadhyay, 2004).

The current approach to cancer treatment revolves around personalized medicine, which entails tailoring treatment strategies based on the unique biological characteristics of individual patients (Voss et al., 2015; Krzyszczyk et al., 2018). Biomarkers and targeted therapeutics play a crucial role in this personalized treatment paradigm, allowing for precise and effective therapy. Biomarkers present in blood, tissues, proteins, and genes can facilitate cancer identification, assess disease progression, monitor treatment response, and predict toxicity. Furthermore, the heterogeneity observed among cancer cells necessitates a deeper understanding of drug resistance mechanisms, which can affect treatment strategies, biomarker selection, and treatment modification (Kamel and Al Amodi, 2016).

In light of these considerations, this study focused on the newly synthesized c-Src inhibitor Si162 and aimed to identify the prognostic biomarkers associated with Si162 response in melanoma cells (Kruewel et al., 2010). By leveraging primary melanoma cells obtained from patients and utilizing gene expression profiling, cytotoxicity assays, and advanced bioinformatic analyses, this study aimed to identify genes that can serve as reliable biomarkers for predicting chemosensitivity to Si162 in melanoma cells (Kruewel et al., 2010).

The specific objectives of this study included culturing primary melanoma cells, treating them with Si162, and assessing their cytotoxic response. Subsequently, the differentially expressed genes between Si162-sensitive and Si162-resistant cells were identified, which allowed for the determination of potential biomarkers associated with Si162 response. Furthermore, this study explored the biological characteristics and pathways associated with Si162 sensitivity and resistance in melanoma cells. Finally, the identified potential biomarkers were validated through quantitative real-time polymerase chain reaction (qRT-PCR) analysis.

Understanding the molecular mechanisms underlying Si162 response and identifying prognostic biomarkers can have profound implications on improving the treatment of melanoma. These findings have the potential to enhance patient stratification, optimize treatment selection, and develop personalized therapeutic approaches. Moreover, investigating the dysregulation of immune-related genes and the involvement of specific pathways, such as epithelial-mesenchymal transition (EMT) and ribosomal protein synthesis, can shed light on the intricate interplay between melanoma cells and the immune system, offering insights into the mechanisms of therapy resistance.

## 2. Materials and methods

### 2.1. Primary melanoma cell culture

The primary melanoma cells used in this study were previously described (Lotem et al., 2002; Lotem et al., 2016). Seven melanoma primary cells (M1, M21, M24, M84, M133, M307, and M2025) were selected from a larger group of over 40 primary melanoma cells obtained from Hadassah Medical University. In order to maintain sample consistency, it was ensured that none of the patients had undergone chemotherapy or radiotherapy before their surgeries. These cell lines, which were classified as stage III cutaneous malignant melanoma metastatic to the regional lymph nodes in accordance with the updated American Joint Committee on Cancer classification, were carefully chosen. All of the cells were obtained from patients who had been diagnosed with melanoma and received treatment at Hadassah Medical Center in Jerusalem. Both the melanoma cells and the corresponding raw microarray-based gene expression data, known as the Melanoma-Luminex data, were provided by Siena University and Hadassah Medical Center.

The cells were cultured in T25 flasks using either Roswell Park Memorial Institute medium (R0883; Sigma-Aldrich Chemical Co., St. Louis, MO, USA) and Dulbecco’s modified eagle’s medium (D6546; Sigma-Aldrich Chemical Co.). The culture medium was supplemented with 20% heat-inactivated phosphate buffered saline (F6178; Sigma-Aldrich Chemical Co.), 2% L-glutamine (Lonza Group AG, Basel, Switzerland), and 1% Pen/Strep (Lonza Group AG). The cells were maintained at 37 °C in a humidified environment with 5% CO_2_.

### 2.2. c-Src/c-Abl inhibitor Si162 and primary melanoma cell treatment

Si162, a pyrazolo[3,4-d]pyrimidine derivative, was synthesized according to the method described by Kruewel et al. (2010), who also described detailed information about the structural and chemical characteristics of Si162.

To assess the cytotoxic response of melanoma cells to Si162, the cells were grown until they reached a specific density of 70%–80%. The cells were then detached using trypsin and seeded onto a sterile 96-well plate at a density of 1000 cells per well. To allow the cells to adhere to the bottom of the plate, they were incubated for 24 h at 37 °C in a 5% CO_2_ environment.

After the 24-h incubation period, 3 wells were treated with 8 different concentrations of Si162, ranging from 0.02 μM to 20 μM, in 10 μL of medium. The treated cells were further incubated for 72 h at 37 °C in an environment with 5% CO_2_. This experimental setup aimed to evaluate the cytotoxic effects of Si162 on the melanoma cells.

### 2.3.Luminescent adenosine triphosphate (ATP) detection for cytotoxicity and calculation of the half-maximal inhibitory concentration (IC_50_) values with the 6M (6 model)

A highly sensitive luminescent ATP detection method was utilized to assess cytotoxicity and growth inhibition in the cells. The experimental procedure involved culturing the cells on a sterile 96-well plate, followed by treatment with 8 different concentrations of Si162 for a duration of 72 h. After the treatment period, the cells were allowed to equilibrate to room temperature over a period of 30 min.

Once the cells reached room temperature, d-Glo reagent (G7572, Promega, Fitchburg, WI, USA) was added to each well. The cells were then incubated with the d-Glo reagent for 10 min at room temperature. Subsequently, a FLUOstar Omega luminometer (BMG Labtech, Ortenberg, Germany) was employed to measure the luminescence signal, which correlates with the concentration of ATP, and provides an indicator of cell viability and growth.

By analyzing the ATP concentration in each well, the corresponding cell viability and growth percentages were determined. Percent cell viability values were calculated for each cell and IC_50_ values were calculated with the R program using the six model (6M), which was defined in our previous study (Turk et al., 2020).

Briefly, 6 different models were derived from the following nonlinear logistic regression function: where Y is the percent growth of the cells, X is the arithmetic drug concentration, a is the percent growth of the cells when the cells are not treated with the drug (control), d is the percent growth of the cells for infinite dose, i.e. a dose for which there is no additional effect when increased, c is the dose corresponding to percent growth exactly between a and d, and b is the Hill slope factor that is used to define the steepness of the curve fitted.

The following were the conditions required for the generation of the 6-models:

1. 3-Parameter model: the curves were fitted without using Hill slope factor b.
2. 3-Parameter top 100 model: the curves were fitted without using Hill slope factor b and with a = 100.
3. 3-Parameter bottom 0 model: the curves were fitted without using Hill slope factor b and with d = 0.
4. 4-Parameter model: the formula was used as it is.
5. 4-Parameter top 100 model: the curves were fitted with a = 100.
6. 4-Parameter bottom 0 model: the curves were fitted with d = 0.

Then, 6 different drug response parameters were calculated out of the fitted curves, as follows:

- IC_50_: Value of X when Ŷ = 50%
- IC_90_: Value of X when Ŷ = 90%
- IC_95_: Value of X when Ŷ = 95%
- EC_50_: Value of X when Ŷ = a + d
- Amax: a − d
- Activity area: ∑ŶX, (sum of Ŷs for each 0.01 increment of X fitted), where Ŷ is the predicted value of Y by the curve fitted.

To calculate the inhibitor exposure variables like the IC_50_, EC_50_, activity area, and Amax, proliferations of the cells were analyzed as a component of the inhibitor levels using nonlinear regression, as described in The National Institutes of Health/NIH Chemical Genomics Center assay guidelines (DeLean et al., 1978). Although the nonlinear regression function used to delineate the inputs is commonly used in cytotoxicity measurements, 6 different models of this algorithm were used herein to calculate the cytotoxicity values and choose the lowest standard error. With the 6M approach, the IC_50_ values for cells treated with Si162 were calculated separately using R script SixModelIC50 V3.r (https://github.com/muratisbilen/6-Model_IC50_CalculationV3.git) (Turk et al., 2020). Thus, 6 different growth inhibition curves for 7 melanoma cells (42 curves in total) were drawn by the SixModelIC50 V3.r R code and the IC_50_ values were calculated. The IC_50_ values with the lowest standard error were selected.

### 2.4. Melanoma gene signature analyses

#### 2.4.1. Dataset, cohorts, and data normalization

The whole genome expression raw data for melanoma cells, specifically the Melanoma-Luminex data, were generously provided by collaborators from Hadassah Medical Center.

To ensure comparability and accurate analysis, the raw data underwent normalization using the robust multi-array average (RMA) method. This normalization process was carried out using the Biometrics Research Branch at the National Cancer Institute (BRB)-Array Tools, which helps to preprocess and analyze microarray data. Normalization is crucial for removing systematic biases and variations across samples.

#### 2.4.2. Identifying differentially expressed genes between the resistant/sensitive groups and linear regression analysis

The IC_50_ values for each melanoma cell line were determined individually for Si162 using the 6M. Based on their susceptibility to Si162, the cells were divided into 2 groups. The sensitive group (M1, M21, M24, and M84) comprised cells with IC_50_ values less than 4 μM, while the resistant group (M133, M2025, and M307) included cells with IC_50_ values greater than 4 μM.

To identify genes that exhibited significant expression differences between the groups, an unpaired t-test analysis was performed (p < 0.01, FC > 1). Genes showing statistically significant alterations in expression variation were determined.

To explore the relationship between the gene expression profiles of all of the cell lines and the sensitivity/resistance profiles of the Si162 IC_50_ data, Pearson correlation analysis was conducted using the Melanoma-Luminex data.

Differentially expressed genes between the resistant and sensitive groups were identified based on the criteria of p < 0.01 and a fold change (FC) >1 cutoff.

#### 2.4.3. Hierarchical clustering, network, and pathway analysis

The objective was to cluster the groups based on their gene expression profiles using hierarchical clustering analysis. To achieve this, Cluster 3.0 (http://bonsai.hgc.jp/~mdehoon/software/cluster/software.htm) was utilized, employing the Euclidean distance as a similarity measure and Complete Linkage as the clustering method (DeHoon et al., 2004). Following the cluster analysis, the data were standardized, and the standardized data were visualized using Treeview (http://jtreeview.sourceforge.net/) (Saldanha, 2004).

To explore the coexpression, gene communication, and pathway networks among the differentially expressed genes within the groups, coexpression, gene interaction, and pathway network analyses were conducted using Cytoscape (Shannon et al., 2003). Coexpression was represented by purple lines, while genetic interaction was represented by green lines between each gene.

The dataset comprised 36 differentially expressed genes. To determine whether the datasets shared genes with similar functions and uncover the roles of distinct gene sets within the network, the datasets were combined, processed, and displayed. The network connections between these genes were identified. To gain insight into the biological relevance of these genes, the Database for Annotation, Visualization, and Integrated Discovery (DAVID) software was employed (Sherman et al., 2021). This facilitated identification of the cellular processes involving the genes.

#### 2.4.4. Gene set enrichment analysis (GSEA)

To investigate the molecular pathways responsible for the distinct responses to Si162 in the sensitive and resistant groups identified by the 36 genes, GSEA was conducted (Subramanian et al., 2005). The objective was to identify gene sets associated with specific pathways within the groups. The data contained probe set IDs, specifically 22,268, which were further reduced to 13,321 genes. In cases where multiple probe sets were available for a gene, the probe set with the highest expression was selected. The analysis focused on gene sets annotated with the same Gene Ontology (GO) term, utilizing the C5 Gene Ontology 6.1 database. GSEA employed default filtering criteria, including gene cluster sizes ranging from 15 to 500, to analyze a total of 5081 gene sets. The analysis identified gene groups that exhibited differential expressions between the different groups. Subsequently, the pathways associated with these genes were determined.

#### 2.4.5. Gene expression profiling via qRT-PCR

To obtain the profiles of the genes identified in the melanoma cells, the following steps were undertaken: RNA extraction from cells, DNA fragmentation, cDNA production, and qRT-PCR experiments.

For the gene expression profiling, a 7500 Real-Time PCR System (Applied Biosystems, San Francisco, CA, USA) and SYBR Green PCR Master Mix for qRT-PCR (Applied Biosystems) were utilized. The PCR procedures followed the recommended cycling conditions provided by the manufacturer. To ensure accuracy, each response was compared to an endogenous standard, glyceraldehyde 3-phosphate dehydrogenase. The PCR primers were designed using the Primer3 Tool (https://primer3.ut.ee/), and the details can be found in Table S1. The gene expression levels were determined using the delta-delta Ct method.

## 3. Results

### 3.1. Pyrazolo[3,4-d]pyrimidine derivative Si162 structure

The chemical structure and properties of the pyrazolo[3,4-d] pyrimidine derivative Si162 were thoroughly described in a previous study (Kumar et al., 2015), which provided a detailed characterization of Si162, including its molecular structure and the allocation of substituents. Additionally, it investigated the effects of Si162 on the enzyme kinetics of c-Abl and c-Src, 2 important signaling proteins involved in cellular processes (Figure S1) (Kumar et al., 2015).

The comprehensive analysis of the chemical structure of Si162 and its interactions with c-Abl and c-Src enzymes presented by Kumar et al. (2015) serves as a foundation for understanding the pharmacological properties and potential therapeutic applications of Si162. This knowledge is crucial for further investigations into the efficacy of Si162 as a targeted therapy for various diseases, particularly in the context of cancer treatment.

### 3.2. Determination of the Si162 cell cytotoxicity and IC_50_ values

**Figure 1:**
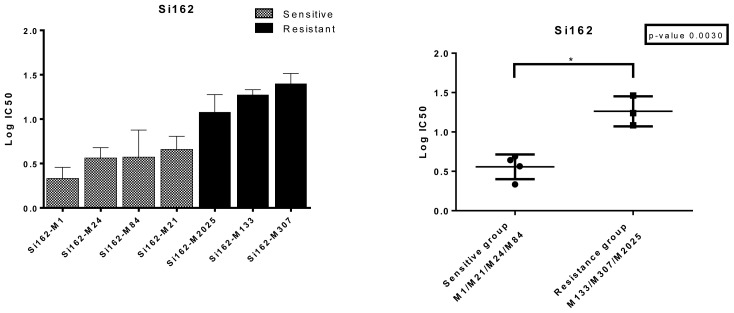
Melanoma cells divided into resistant and sensitive groups based on their IC_50_ values against Si162 (a). There is a statistically significant (p < 0.05, p = 0.0030) difference between the 2 groups in terms of resistance to Si162 (b).

Highly sensitive cell cytotoxicity and growth inhibition assays utilizing the ATP luminescent technique were employed to evaluate the cytotoxic effects of Si162. The IC_50_ values, representing the concentration of Si162 required to inhibit cell growth by 50%, were determined individually for each melanoma cell using the 6M approach (Turk et al., 2020). Input files containing Si162 concentrations and corresponding cell growth percentages were created for each cell and analyzed using the R program. Six different IC_50_ values, along with the EC_50_, activity area, and Amax values, were calculated for each cell (Figure S2). To determine the most representative IC_50_ value, the approach employed was to select the value with the lowest standard error among the 6 calculated IC_50_ values. Thus, the IC_50_ values with the lowest standard error were chosen for further analysis. Figure S2 depicts the growth inhibition curves and corresponding IC_50_ values with the lowest standard errors for each melanoma cell individually, illustrating the variation in response to Si162 cytotoxicity. Based on the individual IC_50_ values, the melanoma cells were classified into 2 distinct groups: the sensitive group (M1, M21, M24, and M84) comprising cells with IC_50_ values below 4 μM, and the resistant group (M133, M2025, and M307) consisting of cells with IC_50_ values greater than 4 μM (Figure 1a). The clear separation of the cells into these 2 groups is evident in Figure 1a. The significant difference (p < 0.05, p = 0.0030) in the IC_50_ values between the sensitive and resistant groups against Si162 demonstrates the varying responsiveness of the melanoma cells to the compound (Figure 1b).

### 3.3. Discovery of differentially expressed genes between Si162 sensitive and resistant cells

The existence of 2 separate groups, one of which is sensitive and the other resistant to Si162, clearly implies that there are underlying biological distinctions among these cells. Consequently, it was aimed to establish a gene signature of chemosensitivity for the novel Src inhibitor, Si162. To achieve this, the gene expression data obtained from the melanoma cells were compared using Luminex technology with the IC_50_ values acquired through the in vitro testing.

Using an unpaired t-test, changes in the gene transcript expression were determined and the 36 most differentially expressed genes among the high IC_50_ (>4 μM) and low IC_50_ (<4 μM) groups in response to Si162 treatment were identified (Table S2). Table S2 presents the 36 genes exhibiting statistically significant (p < 0.01) differential expression values between the groups, along with a high correlation with Si162 sensitivity.

**Figure 2:**
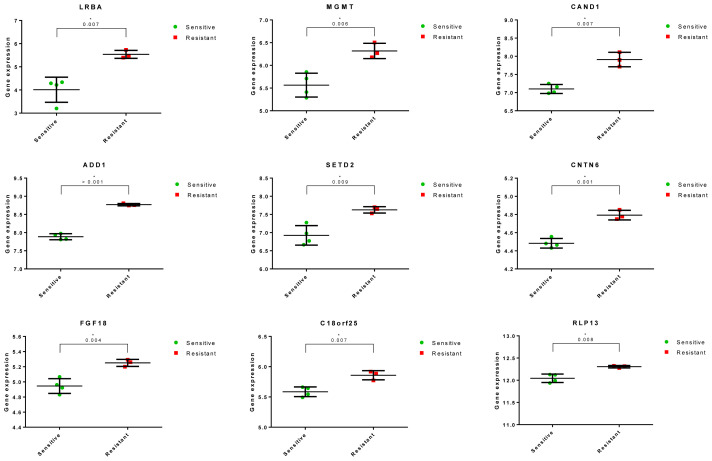
All 9 genes with the potential to be biomarkers for Si162 in melanoma were found to be up-regulated in the resistant group compared to the sensitive group.

**Figure 3:**
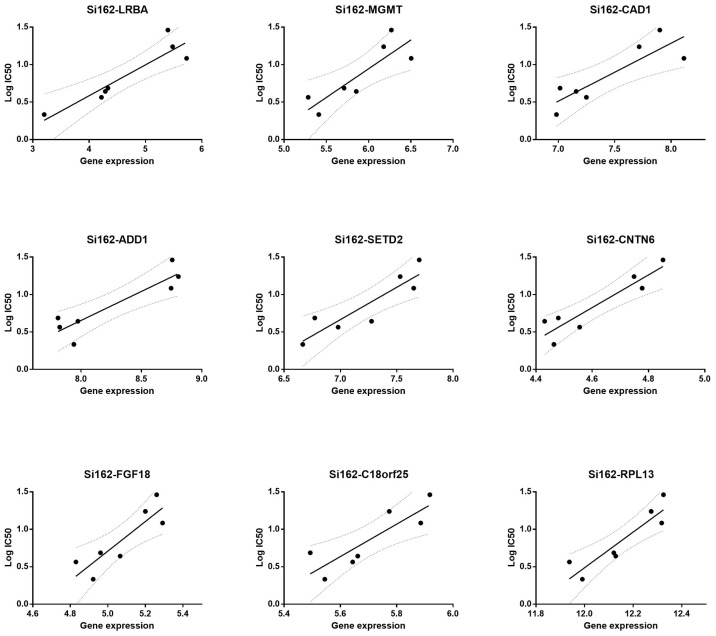
Functional network connectivity between the 36 genes that are differentially expressed between the Si162-resistant and -sensitive groups. Gray dots are genes associated with these genes identified by the program.

Further analysis focused on these 36 genes for biomarker discovery and based on criteria such as a low p-value (<0.01), high r-value (>0.9), and fold change (FC) greater than 1 (>1), 9 genes were selected for validation through qRT-PCR (Table). Figure 2 illustrates the statistically different expression values of these 9 genes between the sensitive and resistant groups, while Figure 3 demonstrates the direct correlation between these genes and the resistance profiles of the melanoma cells treated with Si162.

### 3.4. Biological features of Si162 sensitive and resistant cells

**Figure 4:**
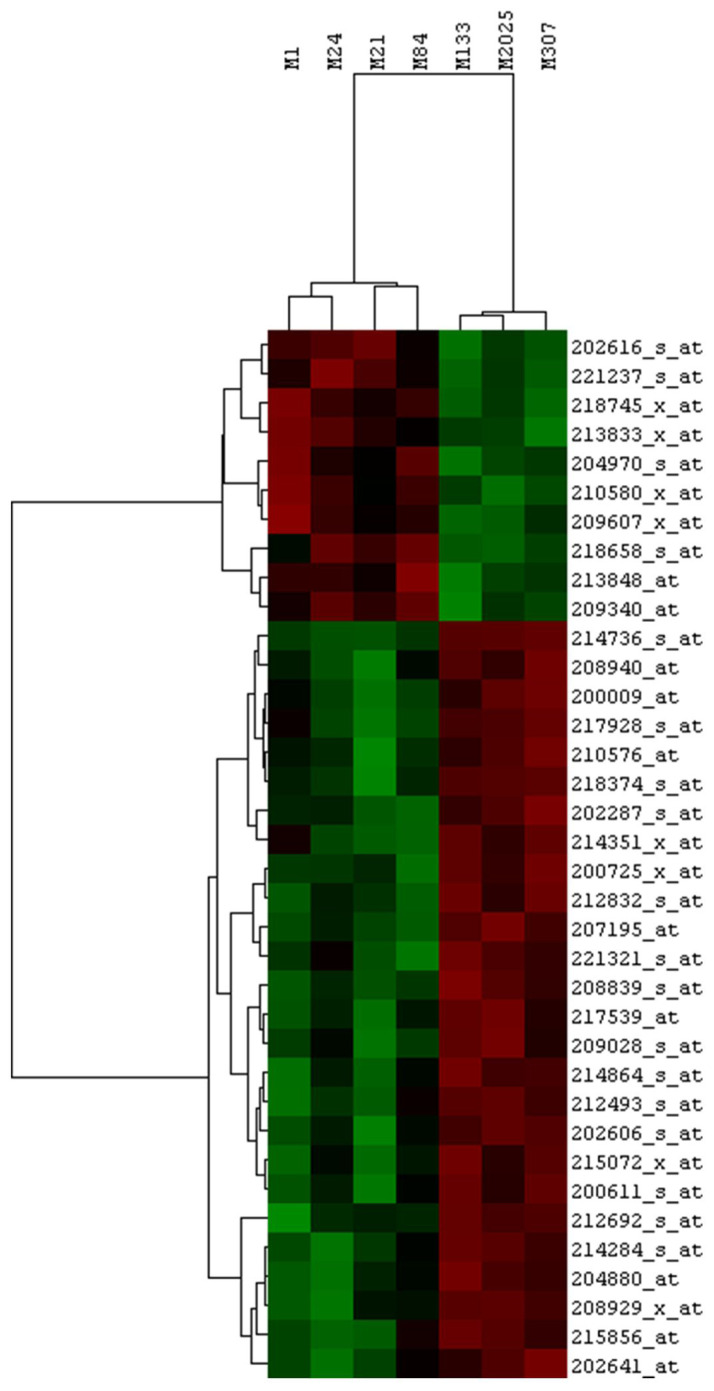
Hierarchical cluster analysis showing that the probes of 36 differentially expressed genes can distinguish well between the Si162-resistant and -sensitive melanoma cells.

The clustering analysis based on the expression of the 36 differentially expressed and highly related genes in the melanoma cells revealed the presence of distinct groups with differential sensitivity to the Si162 treatment (Figure 4).

To gain insight into the interconnectedness of these genes, network analysis was performed. The analysis demonstrated that 33 of the 36 differentially expressed genes exhibited strong coexpression relationships (Figure S3). Additionally, pathway analysis revealed that 9 genes, namely LRBA, MGMT, CAND1, ADD1, SETD2, CNTN6, FGF18, C18orf25, and RPL13, were associated with important biological pathways (Table S3).

GSEA was conducted to evaluate the molecular pathways responsible for the variable responsiveness to Si162 in the sensitive and resistant groups of the melanoma cell lines. The analysis utilized whole-genome expression microarray data encompassing all of the genes expressed in the melanoma cells. The results identified gene sets that exhibited significant (p < 0.05, FDR < 25%) differences between the 2 groups, shedding light on the specific gene clusters associated with these genes (Table S4). Notably, the GSEA revealed a significant enrichment (p < 0.05, FDR < 25%) of immune response-related genes in the resistant group, suggesting a potential dysregulation of the immune response (Figure S4).

### 3.5. In vitro validation of potentially prognostic biomarker genes via qRT-PCR

**Figure 5:**
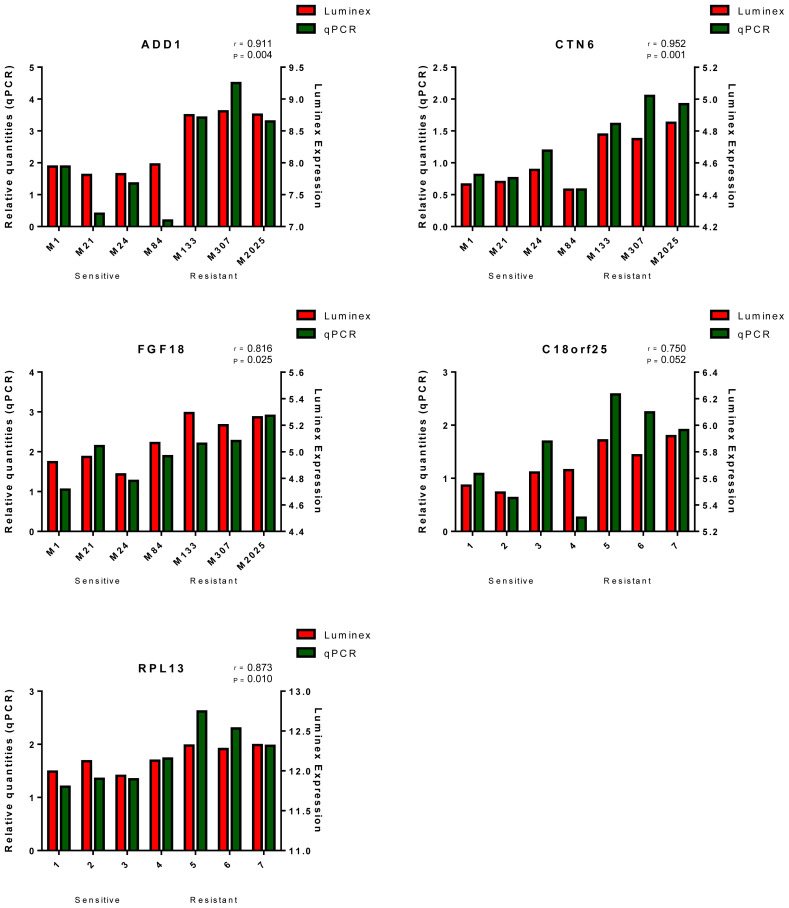
Real-time PCR results present the actual expression rates of the 5 validated biomarker genes for Si162 in the AML cells.

To validate the expression correlation with Si162 resistance, 9 genes, including LRBA, MGMT, CAND1, ADD1, SETD2, CNTN6, FGF18, C18orf25, and RPL13, were selected for in vitro validation. The qRT-PCR analysis was performed to quantify the expression of these genes in 4 sensitive melanoma cells (M1, M21, M24, and M84) and 3 resistant melanoma cells (M133, M307, and M2025). Quantification of the gene expression levels revealed that 5 of these genes, namely ADD1, CNTN6, FGF18, C18orf25, and RPL13, exhibited a relatively higher expression in the resistant cells compared to the sensitive cells (Figure 5). These findings were consistent with the correlation observed between the gene expression levels determined using the Luminex data and the results obtained from the qRT-PCR analysis, further supporting the validity of the gene expression patterns associated with Si162 resistance in melanoma cells (Figure 5).

## 4. Discussion

The findings of this study shed light on the potential of Si162, a novel c-Src inhibitor, in suppressing tumor growth in melanoma cells. However, despite the initial clinical responses observed in targeted therapies and immunotherapies for melanoma, the development of resistance remains an important challenge (Eddy et al., 2020). This highlights the ongoing need for the development of innovative treatment approaches to achieve more successful outcomes in melanoma patients.

The Src family kinases, including c-Src, play a crucial role in promoting cell proliferation, survival, motility, invasiveness, and angiogenesis (Lieu and Kopetz, 2010). The results indicated that the Src pathway is associated with pathways related to EMT, TGF-β signaling, and ribosomal protein synthesis. These findings suggest that the Src pathway may intersect with these pathways, contributing to the development of resistance mechanisms in melanoma cells.

Through a comparative analysis of the Si162-resistant and Si162-sensitive groups, 9 genes were identified that exhibited significant differential expression (p < 0.01, FC > 1). Among these genes, CNTN6, ADD1, and FGF18 were previously associated with EMT in solid tumors, while ARKL1 (C18orf25) was implicated in the regulation of TGF-β-negative regulators (CNTN6) (Inoue and Imamura, 2008; Niessen et al., 2008; Bisogno et al., 2018; Song et al., 2018). Additionally, higher expression levels of RPL13, a ribosomal protein, were observed in the Si162-resistant group, indicating its potential involvement in cancer cell growth through protein synthesis regulation (Kobayashi et al., 2006).

The gene set enrichment analysis revealed the enrichment of adaptive and innate immune system-related gene sets in the Si162-resistant group, suggesting the dysregulation of immune-related genes in these cells. This finding highlights the complex interplay between cancer cells and the immune system, as cancer cells employ various strategies to evade immune detection and dampen immune responses (Eddy et al., 2020). Epigenetic dysregulation of immune-related pathways in melanoma further contributes to altered gene expression and impacts the response to chemotherapy components (Eddy et al., 2020).

The findings of this study have important implications for the future perspective and usage of Si162 in the treatment of melanoma. Despite the initial clinical responses observed in targeted therapies and immunotherapies, the development of resistance remains a major challenge in melanoma treatment. The identification of Si162 as a potent inhibitor of c-Src, a key player in promoting melanoma cell growth and survival, suggests its potential as an effective therapeutic agent. One future perspective is the clinical translation of Si162 for the treatment of melanoma patients, supported by the preclinical evidence demonstrating its ability to suppress tumor growth. The potential of Si162 to overcome drug resistance mechanisms, as indicated by its impact on the Src pathway and association with pathways related to EMT, TGF-β signaling, and ribosomal protein synthesis, opens new avenues for therapeutic interventions. Additionally, the identification of biomarkers associated with Si162 resistance, such as CNTN6, ADD1, FGF18, C18orf25, and RPL13, offers the opportunity for personalized treatment strategies and the development of combination therapies to overcome resistance. Further investigation into immune dysregulation in Si162-resistant melanoma cells and the exploration of immune-based combination therapies are essential for enhancing treatment efficacy. In conclusion, Si162 and related therapeutic approaches hold promise for addressing drug resistance and improving outcomes in melanoma patients through personalized treatment strategies and immune modulation.

In conclusion, this study provides insight into the potential mechanisms underlying Si162 resistance in melanoma cells and identified 5 genes (CNTN6, ADD1, FGF18, C18orf25, and RPL13) as potential prognostic biomarkers for Si162 resistance. Understanding the biological pathways and molecular mechanisms involved in resistance can guide the development of novel therapeutic strategies to overcome drug resistance and improve treatment outcomes in melanoma patients. Furthermore, targeting immune-related pathways affected by epigenetic dysregulation may hold promise for enhancing the efficacy of therapies in melanoma. Future studies focusing on the validation and functional characterization of these biomarkers and the exploration of combination therapies are warranted to advance the field of melanoma treatment.

## Supplementary Data

Supplementary Figure 1 Chemical structure and chemical properties of Si162 component.

Supplementary Figure 2 Melanoma cell’s IC50 values against Si162, 6 different non-linear regression algorithms (6 model) were used to calculate the IC50, the model with the lowest standard error was chosen.

Supplementary Figure 3 Functional network connectivity between 36 genes that are differentially expressed between Si162 resistant and sensitive groups. Gray dots are genes associated with these genes identified by the program.

Supplementary Figure 4 Leukocyte-mediated immunity (left) and adaptive immune response related immunoglobulin superfamily related genesets are among significantly enriched at FDR < 25% in the Si362 resistant group.

## Figures and Tables

**Table:** The 9 genes with a fold change >1 and Pearson r > 0.8 selected for in vitro validation.

Gene	T test	FC (R/S)	r-value	p-value
LRBA	0.007207	1.380658	0.932858	0.002163
MGMT	0.005927	1.135193	0.897511	0.006108
CAND1	0.007251	1.114019	0.919897	0.00334
ADD1	3.5E - 05	1.111973	0.895198	0.00645
SETD2	0.009224	1.101595	0.914935	0.00387
CNTN6	0.001001	1.069136	0.933926	0.00208
FGF18	0.003809	1.061887	0.902283	0.005436
C18orf25	0.007107	1.048878	0.905983	0.004946
RPL13	0.008296	1.021695	0.926089	0.00274
