# Supplementary figures and images for: Prognostic gene biomarkers for c-Src inhibitor Si162 sensitivity in melanoma cells
Source: Turk J Biol. 2023 Nov 6;48(1):13–23. doi: 10.55730/1300-0152.2678 (PMC11042866; doi:10.55730/1300-0152.2678)

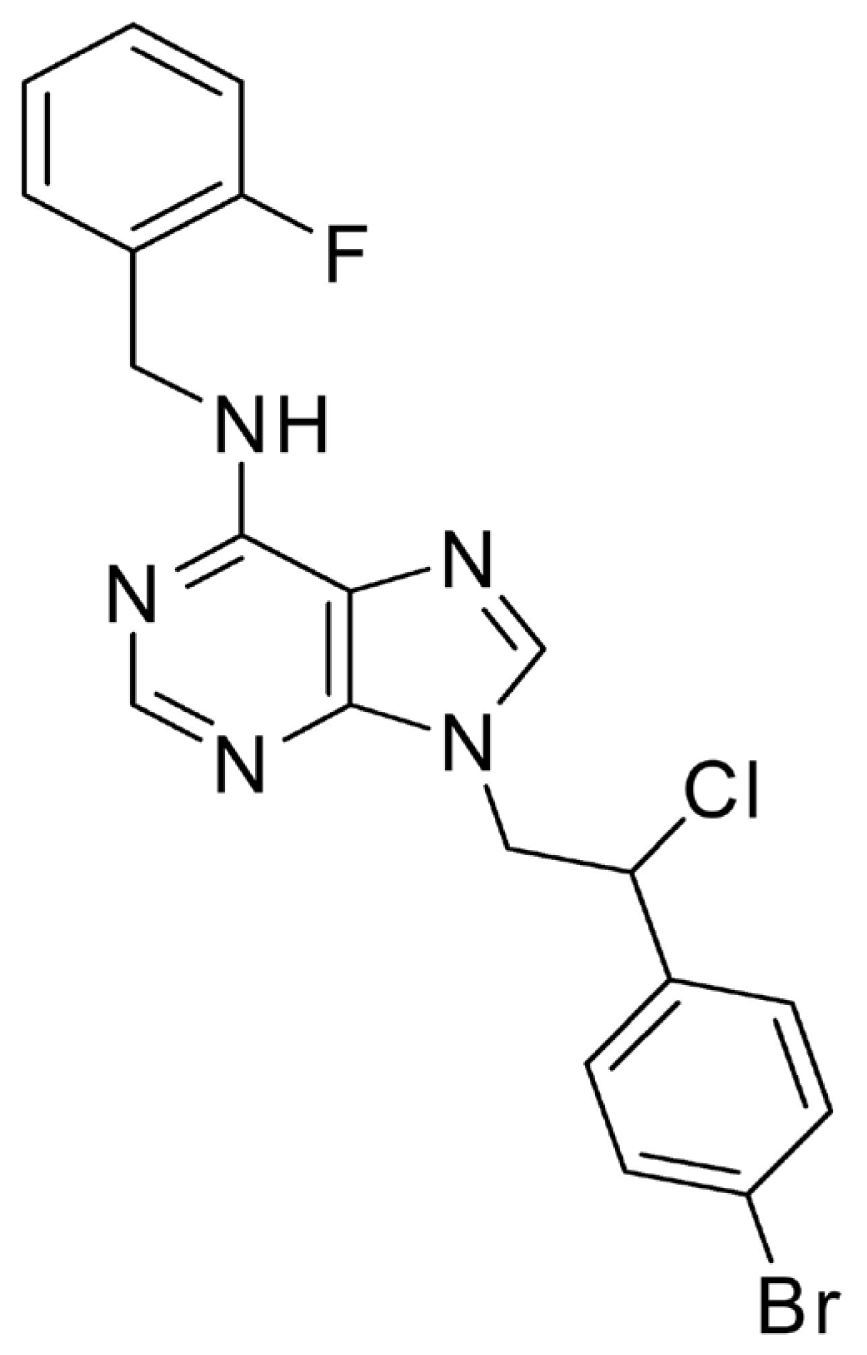

Supplement: Supplementary Figure 1 — Chemical structure and chemical properties of Si162 component. [file tjb-48-01-013s1.tif]

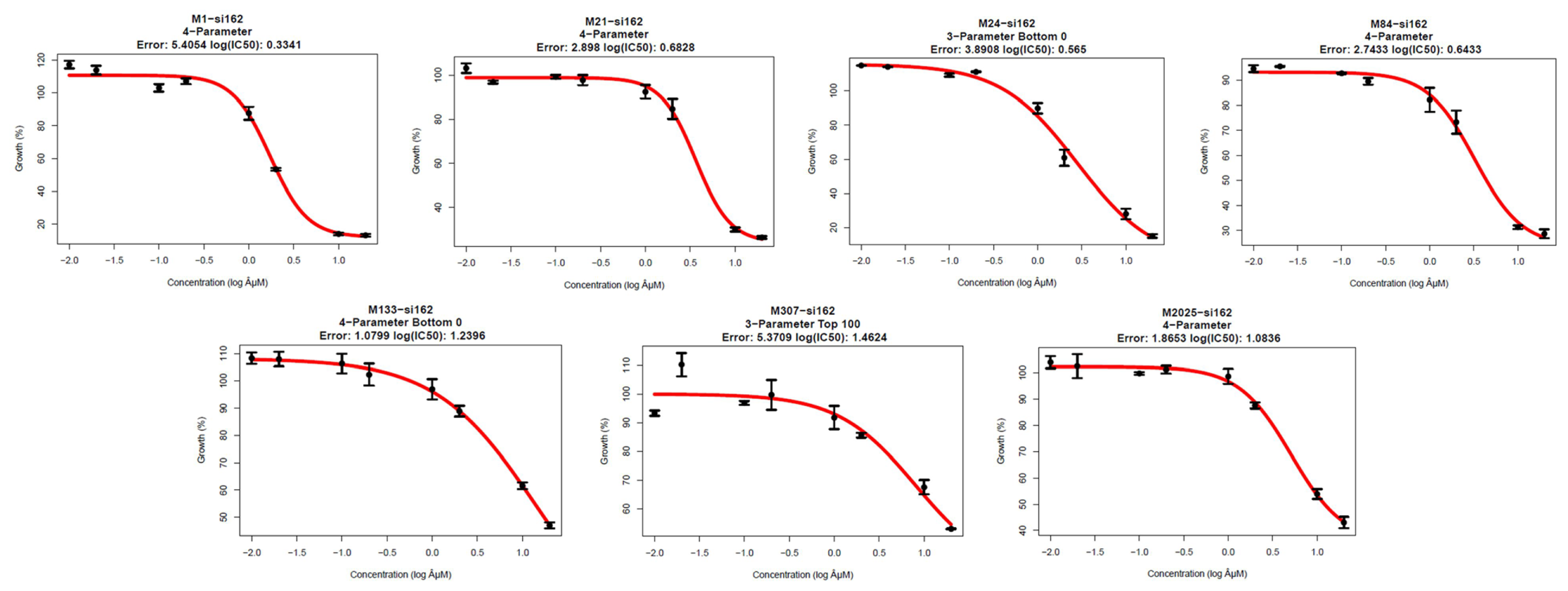

Supplement: Supplementary Figure 2 — Melanoma cell’s IC50 values against Si162, 6 different non-linear regression algorithms (6 model) were used to calculate the IC50, the model with the lowest standard error was chosen. [file tjb-48-01-013s2.tif]

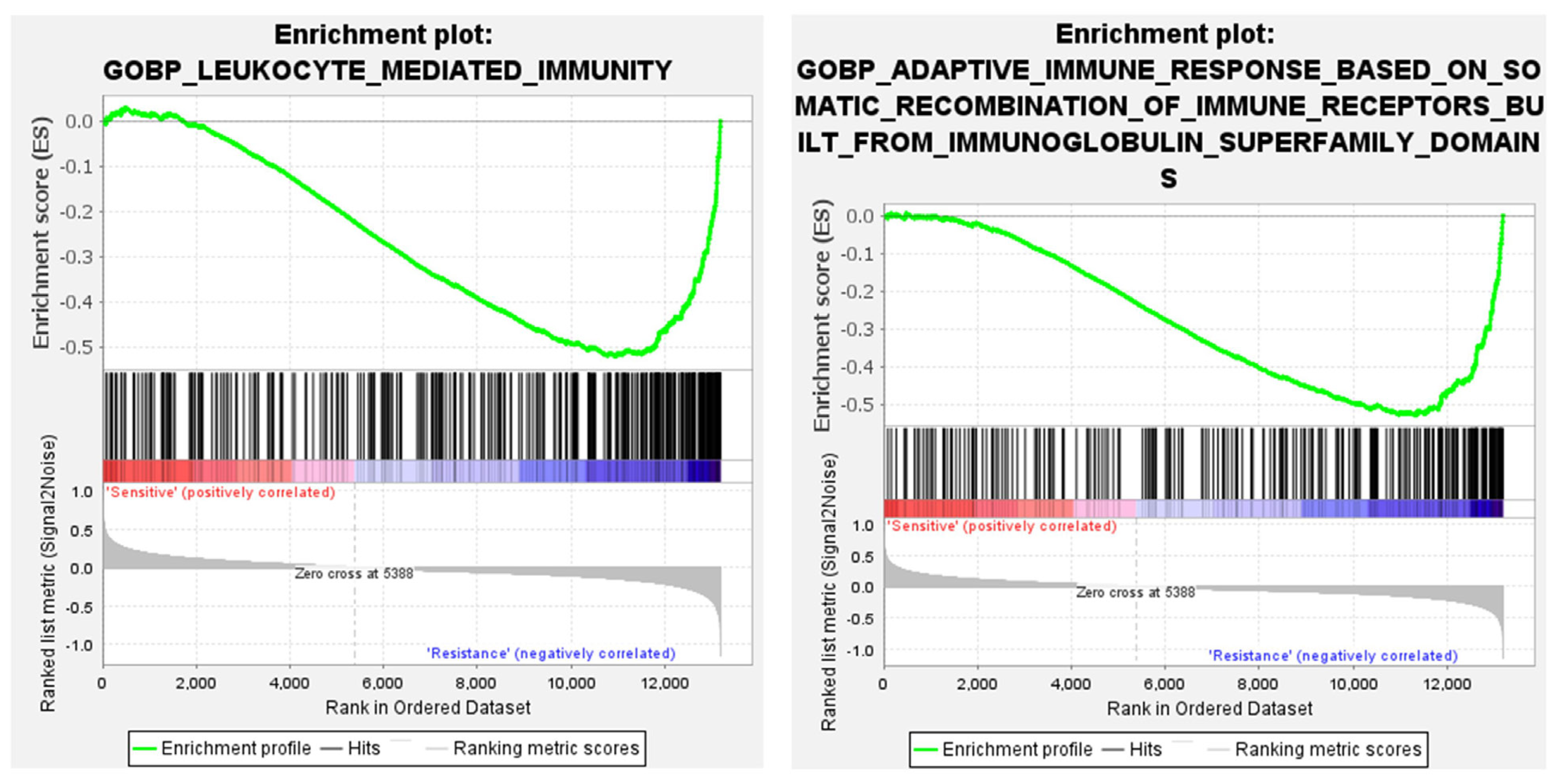

Supplement: Supplementary Figure 3 — Functional network connectivity between 36 genes that are differentially expressed between Si162 resistant and sensitive groups. Gray dots are genes associated with these genes identified by the program. [file tjb-48-01-013s3.tif]

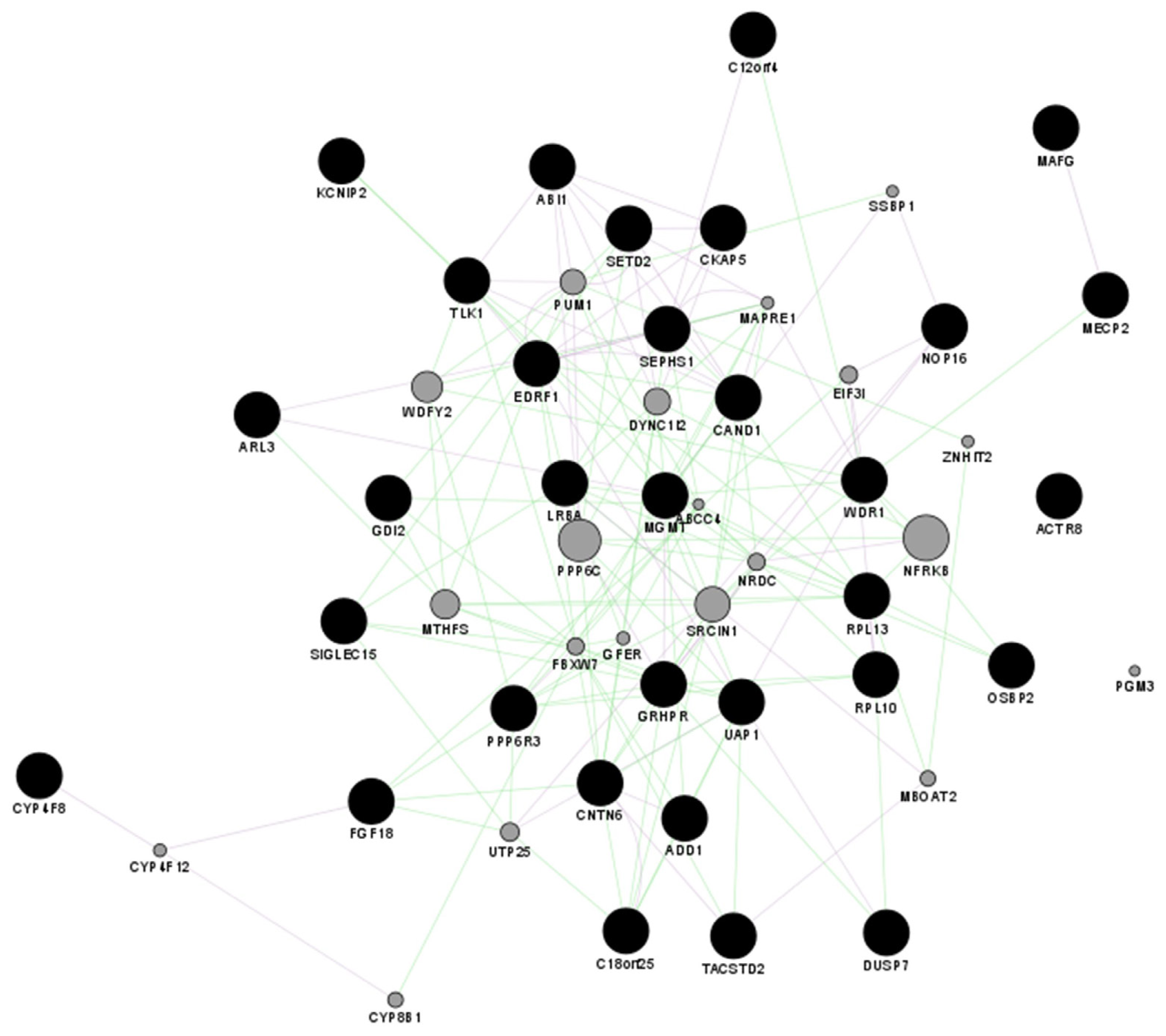

Supplement: Supplementary Figure 4 — Leukocyte-mediated immunity (left) and adaptive immune response related immunoglobulin superfamily related genesets are among significantly enriched at FDR < 25% in the Si362 resistant group. [file tjb-48-01-013s4.tif]
