# Supplementary material for: Prognostic gene biomarkers for c-Src inhibitor Si162 sensitivity in melanoma cells
Source: Turk J Biol. 2023 Nov 6;48(1):13–23. doi: 10.55730/1300-0152.2678 (PMC11042866; doi:10.55730/1300-0152.2678)
Supplement: Supplementary file 5 [file BIY-2305-20_1_Supplementary_Table_1.docx]

| **Gene Name** | **Primers** | |
| --- | --- | --- |
|  | Forward | Reverse |
| CNTN6 | GCCTGGCTGAGAGCTTGAA | TCCCCCATCACACCATCAGT |
| ADD1 | CCACGATTCGCTTCTGAGGA | TCTTCACAGAAAGCAGGGCT |
| ARKL1 | AGGTGTTCTGGGCATCTGAA | CCAGCTATGAAAGACGGCAC |
| FGF18 | AAAGTAGTACTCCGCCCACC | CGATAGCAGCATCCTCCAGA |
| RPL13 | TCCCCATGTCTTCTGTGTCC | TCACCTATTTCCACGCCAGT |
| LRBA | TTGTTTCCTGCCAGCTTCAC | CAGCCTCTCTCAACACAGGA |
| MGMT | CCGAGGCCTGGATGAAAATG | CGCTGTGTGACTTATCCTGC |
| CAND1 | CTGTTGCCTGGACTGGAGTA | GCCAACACAGGATTGCTTGA |
| SETD2 | TACACCAAGACTCCAGCGTT | ACTTGGCTGGGCATAACTCT |
| GAPDH | CCAGAACATCATCCCTGCCT | CCTGCTTCACCACCTTCTTG |

**Supplementary Table 1.** Primer sequences for discovered potential biomarker genes
