# Supplementary material for: Prognostic gene biomarkers for c-Src inhibitor Si162 sensitivity in melanoma cells
Source: Turk J Biol. 2023 Nov 6;48(1):13–23. doi: 10.55730/1300-0152.2678 (PMC11042866; doi:10.55730/1300-0152.2678)
Supplement: Supplementary file 6 [file BIY-2305-20_1_Supplementary_Table_2.docx]

**Supplementary Table 2.** 36 genes differentially expressed (p-value<0.01) between resistant and sensitive groups to Si162

| **Gene** | **T.Test p value** | **FC (R/S)** | **Pearson R value** | **Pearson P value** |
| --- | --- | --- | --- | --- |
| SULT1A3 /// SULT1A4 | 0,007066 | 0,8815 | -0,96327 | 0,000487 |
| SULT1A3 /// SULT1A4 | 0,009862 | 0,874543 | -0,96083 | 0,000571 |
| CNTN6 | 0,001001 | 1,069136 | 0,933926 | 0,00208 |
| LRBA | 0,007207 | 1,380658 | 0,932858 | 0,002163 |
| RPL13 | 0,008296 | 1,021695 | 0,926089 | 0,00274 |
| CAND1 | 0,007251 | 1,114019 | 0,919897 | 0,00334 |
| SETD2 | 0,009224 | 1,101595 | 0,914935 | 0,00387 |
| C18orf25 | 0,007107 | 1,048878 | 0,905983 | 0,004946 |
| MAFG | 0,007825 | 0,87343 | -0,90251 | 0,005404 |
| FGF18 | 0,003809 | 1,061887 | 0,902283 | 0,005436 |
| TMEM161A | 0,002563 | 0,951082 | -0,89965 | 0,0058 |
| MGMT | 0,005927 | 1,135193 | 0,897511 | 0,006108 |
| ADD1 | 3,5E-05 | 1,111973 | 0,895198 | 0,00645 |
| GRHPR | 0,006017 | 1,110608 | 0,86478 | 0,011991 |
| NOP16 | 0,006826 | 0,970439 | -0,86192 | 0,012615 |
| ABI1 | 0,009265 | 1,125272 | 0,858569 | 0,013369 |
| SIGLEC15 | 0,008369 | 1,201427 | 0,855781 | 0,014015 |
| TLK1 | 0,009825 | 1,114181 | 0,833991 | 0,01968 |
| CKAP5 | 0,004199 | 1,058192 | 0,828458 | 0,021294 |
| MECP2 | 0,002197 | 0,907695 | -0,81815 | 0,024494 |
| ARL3 | 0,008517 | 1,131606 | 0,809882 | 0,027245 |
| RPL10 | 0,002034 | 1,017869 | 0,804595 | 0,02909 |
| DUSP7 | 0,008348 | 0,872423 | -0,79938 | 0,030979 |
| C10orf137 | 0,008183 | 1,058184 | 0,791945 | 0,033783 |
| KCNIP2 | 0,00802 | 1,047082 | 0,781026 | 0,03815 |
| C12orf4 | 0,008061 | 1,124106 | 0,772023 | 0,041973 |
| UAP1 | 0,009432 | 0,903066 | -0,7656 | 0,044823 |
| OSBP2 | 0,006009 | 0,905876 | -0,76449 | 0,04533 |
| ACTR8 | 0,007841 | 0,9776 | -0,7635 | 0,045777 |
| TACSTD2 | 0,004031 | 1,051553 | 0,759646 | 0,04756 |
| WDR1 | 0,009066 | 1,089048 | 0,754989 | 0,049762 |
| GDI2 | 0,005278 | 1,171652 | 0,728563 | 0,063294 |
| SEPHS1 | 0,007277 | 1,187406 | 0,697561 | 0,081432 |
| CYP4F8 | 0,008 | 1,194932 | 0,69386 | 0,083761 |
| SAPS3 | 0,009186 | 1,04489 | 0,683719 | 0,09032 |
| RPL13 | 0,008669 | 1,022445 | 0,63739 | 0,123601 |
