# Supplementary material for: Prognostic gene biomarkers for c-Src inhibitor Si162 sensitivity in melanoma cells
Source: Turk J Biol. 2023 Nov 6;48(1):13–23. doi: 10.55730/1300-0152.2678 (PMC11042866; doi:10.55730/1300-0152.2678)
Supplement: Supplementary file 7 [file BIY-2305-20_1_Supplementary_Table_3.docx]

**Supplementary Table 3**. Pathway analysis of 8 out of 9 differentially expressed genes.

| **50S ribosomal protein L13, chloreplastic (RPL13)** |
| --- |
| translation, |
| ribosome, |
| structural constituent of ribosome, |
| Ribosomal protein L13, Ribosomal protein L13, bacterial-type, Ribosomal protein L13, conserved site, |
| **6-0-methylguanine-DNA methyltransferase(mgmt)** |
| DNA dealkylation involved in DNA repair, methylation, |
| methylated-DNA- protein -cysteine S- methyltransferase activity, |
| Methylated-DNA-[protein]-cysteine S-methyltransferase, active site, Methylguanine DNA methyltransferase, ribonuclease-like, Methylated-DNA-[protein]-cysteine S-methyltransferase, DNA binding |
| DNA damage, DNA repair, |
| Methyltransferase, Transferase, |
| COMPBIAS: Basic and acidic residues, DOMAIN: DNA_ binding DOMAIN: Methyltransf1N, REGION: Disordered. |
| **ADDA protein (Add1)** |
| cytoskeleton, |
| Class II aldolase/adducin N-terminal |
| SM01007, |
| COMPBIAS: Basic and acidic residues, COMPBIAS: Polar residues: Aldolase REGION: Disordered |
| **Cis-aconitate decarboxylase(cad1)** |
| lyase activity, |
| MmgE/PrpD, |
| DOMAIN: MmgE_PrpD, DOMAIN: MmgE_ PrpD_C |
| **Contactin 6(CNTNG)** |
| cell adhesion, axon guidance, |
| plasma membrane, |
| Notch binding, |
| Immunoglobulin subtype 2, Immunoglobulin subtype, Fibronectin, type III, Immunoglobulin-like domain, Immunoglobulin I-set, Immunoglobulin-like fold, |
| FN3, IGc2, IG, |
| Immunoglobulin domain, Repeat, |
| DOMAIN: Fibronectin type-IlI, DOMAIN: Ig-like, REGION: Disordered |
| **Fibroblast growth factor (Fgf18)** |
| Fibroblast growth factor receptor signaling pathway, positive regulation of cell division |
| extracellular region |
| Fibroblast growth factor receptor binding, growth factor activity |
| Heparin-binding growth factor/Fibroblast growth factor, Cytokine, IL-1-like |
| FGF |
| Mitogen |
| **Histone-lysineN-methyltransferaseSETD2(Setd2).** |
| regulation of transcription, DNA-templated, histone H3-K36 trimethylation, |
| nucleus, chromosome, |
| histone methyltransferase activity (H3-K36 specific), |
| WW domain, SRI, Set2 Rpb1 interacting, |
| WW, |
| Nucleus, |
| Methyltransferase, Transferase, |
| COMPBIAS: Basic and acidic residues, COMPBIAS: Polar residues, COMPBIAS:Pro residues, DOMAIN: WW, REGION: Disordered, |
| **LPS responsive beige-like anchor protein (LRBA)** |
| protein localization, |
| cytosol, membrane, integral component of membrane, |
| protein kinase binding, |
| BEACH domain, WD40 repeat, Domain of unknown function DUF1088, Armadillo-like helical, Pleckstrin homology-like domain, Concanavalin A-like lectin/glucanase, subgroup, WD40/YVTN repeat-like-containing domain, Armadillo-type fold, PH-BEACH domain, |
| WD40, SM01026, |
| Membrane, |
| Transmembrane, WD repeat, Transmembrane helix, |
| COMPBIAS: Polar residues, DOMAIN: BEACH, DOMAIN: BEACH-type PH, REGION: Disordered, REPEAT: WD, TRANSMEM: Helical, |
