# Supplementary material for: Prognostic gene biomarkers for c-Src inhibitor Si162 sensitivity in melanoma cells
Source: Turk J Biol. 2023 Nov 6;48(1):13–23. doi: 10.55730/1300-0152.2678 (PMC11042866; doi:10.55730/1300-0152.2678)
Supplement: Supplementary file 8 [file BIY-2305-20_1_Supplementary_Table_4.docx]

| **GS** | **SIZE** | **ES** | **NES** | **NOM p-val** | **FDR q-val** | **FWER p-val** | **RANK AT MAX** |
| --- | --- | --- | --- | --- | --- | --- | --- |
| HP_DECREASED_CIRCULATING_IGG_LEVEL | 57 | -0,68 | -2,4 | 0 | 0 | 0 | 2677 |
| GOBP_B_CELL_RECEPTOR_SIGNALING_PATHWAY | 51 | -0,7 | -2,38 | 0 | 0 | 0 | 1064 |
| HP_ABNORMAL_CIRCULATING_IGM_LEVEL | 57 | -0,67 | -2,36 | 0 | 0 | 0 | 1813 |
| GOBP_LYMPHOCYTE_MEDIATED_IMMUNITY | 246 | -0,55 | -2,32 | 0 | 0 | 0 | 2423 |
| HP_ABNORMAL_CIRCULATING_IGA_LEVEL | 62 | -0,64 | -2,31 | 0 | 0 | 0 | 1813 |
| HP_ABNORMAL_CIRCULATING_IGG_LEVEL | 68 | -0,64 | -2,31 | 0 | 0 | 0 | 2677 |
| HP_ABNORMAL_B_CELL_MORPHOLOGY | 44 | -0,69 | -2,29 | 0 | 0 | 0 | 1813 |
| HP_DECREASED_CIRCULATING_IGA_LEVEL | 41 | -0,69 | -2,28 | 0 | 0 | 0 | 1813 |
| GOBP_IMMUNE_RESPONSE_REGULATING_CELL_SURFACE_RECEPTOR_SIGNALING_PATHWAY | 213 | -0,54 | -2,27 | 0 | 0 | 0 | 2329 |
| GOBP_LEUKOCYTE_MEDIATED_IMMUNITY | 328 | -0,52 | -2,27 | 0 | 0 | 0 | 2237 |
| HP_ABNORMAL_T_CELL_MORPHOLOGY | 66 | -0,63 | -2,26 | 0 | 0 | 0 | 1929 |
| HP_DECREASED_CIRCULATING_TOTAL_IGM | 33 | -0,72 | -2,26 | 0 | 0 | 0 | 1813 |
| GOBP_ADAPTIVE_IMMUNE_RESPONSE_BASED_ON_SOMATIC_RECOMBINATION_OF_IMMUNE_RECEPTORS_BUILT_FROM_IMMUNOGLOBULIN_SUPERFAMILY_DOMAINS | 252 | -0,53 | -2,25 | 0 | 0 | 0 | 1844 |
| GOBP_B_CELL_MEDIATED_IMMUNITY | 126 | -0,56 | -2,25 | 0 | 0 | 0 | 2225 |
| HP_ABNORMAL_T_CELL_SUBSET_DISTRIBUTION | 51 | -0,65 | -2,24 | 0 | 0 | 0 | 1813 |
| GOBP_ANTIGEN_RECEPTOR_MEDIATED_SIGNALING_PATHWAY | 144 | -0,56 | -2,23 | 0 | 0 | 0 | 2010 |
| HP_IMPAIRED_ANTIGEN_SPECIFIC_RESPONSE | 29 | -0,73 | -2,23 | 0 | 0 | 0 | 2524 |
| GOBP_POSITIVE_REGULATION_OF_LEUKOCYTE_MEDIATED_IMMUNITY | 113 | -0,56 | -2,19 | 0 | 0 | 0,001 | 2225 |
| GOBP_ADAPTIVE_IMMUNE_RESPONSE | 382 | -0,5 | -2,19 | 0 | 0 | 0,001 | 2108 |
| HP_LYMPHOMA | 97 | -0,57 | -2,16 | 0 | 0 | 0,01 | 2557 |

**Suplementary Table 4.** Gene sets were significantly enriched at FDR < 25% in the Si362 resistant group compared to the sensitive group.
